# Comparability of Patients in Trials of eHealth and Face-to-Face Psychotherapeutic Interventions for Depression: Meta-synthesis

**DOI:** 10.2196/36978

**Published:** 2022-09-14

**Authors:** Vera Aemissegger, Jesus Lopez-Alcalde, Claudia M Witt, Jürgen Barth

**Affiliations:** 1 Institute for Complementary and Integrative Medicine University Hospital Zurich and University of Zurich Zurich Switzerland; 2 Faculty of Health Sciences Universidad Francisco de Vitoria Madrid Spain; 3 Unidad de Bioestadística Clínica, Hospital Universitario Ramón y Cajal IRYCIS, CIBERESP Madrid Spain; 4 Berlin Institute of Health, Institute of Social Medicine, Epidemiology and Health Economics Charité – Universitätsmedizin Berlin, corporate member of Freie Universität Berlin, Humboldt-Universität zu Berlin Berlin Germany; 5 Center for Integrative Medicine University of Maryland School of Medicine Baltimore, MD United States

**Keywords:** depression, mental health, digital intervention, eHealth, web-based, randomized controlled trial, RCT, meta-analysis, epidemiology, epidemiological, depressive disorder, mental illness, mental condition, mental disorder, psychotherapy, psychotherapeutic intervention, CBT, iCBT, cognitive behavioral therapy, face-to-face, cognitive therapy, interpersonal therapy

## Abstract

**Background:**

Depressive disorders (DDs) are a public health problem. Face-to-face psychotherapeutic interventions are a first-line option for their treatment in adults. There is a growing interest in eHealth interventions to maximize accessibility for effective treatments. Thus, the number of randomized controlled trials (RCTs) of eHealth psychotherapeutic interventions has increased, and these interventions are being offered to patients. However, it is unknown whether patients with DDs differ in internet-based and face-to-face intervention trials. This information is essential to gain knowledge about eHealth trials’ external validity.

**Objective:**

We aimed to compare the baseline characteristics of patients with DDs included in the RCTs of eHealth and face-to-face psychotherapeutic interventions with a cognitive component.

**Methods:**

In this meta-epidemiological study, we searched 5 databases between 1990 and November 2017 (MEDLINE, Embase, PsycINFO, Google Scholar, and the database of Cuijpers et al). We included RCTs of psychotherapeutic interventions with a cognitive component (eg, cognitive therapy, cognitive behavioral therapy [CBT], or interpersonal therapy) delivered face-to-face or via the internet to adults with DDs. Each included study had a matching study for predefined criteria to allow a valid comparison of characteristics and was classified as a face-to-face (CBT) or eHealth (internet CBT) intervention trial. Two authors selected the studies, extracted data, and resolved disagreements by discussion. We tested whether predefined baseline characteristics differed in face-to-face and internet-based trials using a mixed-effects model and testing for differences with *z* tests (statistical significance set at .05). For continuous outcomes, we also estimated the difference in means between subgroups with 95% CI.

**Results:**

We included 58 RCTs (29 matching pairs) with 3846 participants (female: n=2803, 72.9%) and mean ages ranging from 20-74 years. White participants were the most frequent (from 63.6% to 100%). Other socioeconomic characteristics were poorly described. The participants presented DDs of different severity measured with heterogeneous instruments. Internet CBT trials had a longer depression duration at baseline (7.19 years higher, CI 95% 2.53-11.84; 10.0 vs 2.8 years; *P*=.002), but the proportion of patients with previous depression treatment was lower (24.8% vs 42%; *P*=.04). Subgroup analyses found no evidence of differences for the remaining baseline characteristics: age, gender, education, living area, depression severity, history of depression, actual antidepressant medication, actual physical comorbidity, actual mental comorbidity, study dropout, quality of life, having children, family status, and employment. We could not compare proficiency with computers due to the insufficient number of studies.

**Conclusions:**

The baseline characteristics of patients with DDs included in the RCTs of eHealth and face-to-face psychotherapeutic interventions are generally similar. However, patients in eHealth trials had a longer duration of depression, and a lower proportion had received previous depression treatment, which might indicate that eHealth trials attract patients who postpone earlier treatment attempts.

**Trial Registration:**

PROSPERO CRD42019085880; https://tinyurl.com/4xufwcyr

## Introduction

Depressive disorders (DDs) affect more than 300 million people worldwide and have prevailed as a leading nonfatal health issue for almost 3 decades [1,2]. Their incidence and burden are expected to rise in the future. At present, DDs cause 15% of the days lived with disability and is associated with suicide; a high burden in personal, family, social, and work life; and elevated health care costs [2,3]. Increasing the accessibility to effective interventions for DDs is an international priority [4,5].

Psychotherapeutic interventions aim to improve depressive symptoms by increasing self-efficacy, developing coping skills, and changing cognitions, emotions, and behaviors with exercises and sometimes homework between sessions. Examples include cognitive therapy, cognitive behavioral therapy (CBT), interpersonal therapy, and psychodynamic treatments. Face-to-face psychotherapeutic interventions are accepted as a first-line treatment for DDs [6-9], and the different approaches have benefits from small to large magnitude compared to usual care (standardized mean differences [SMDs] ranging from –0.32 to –0.92) [10,11].

Internet-based (also known as eHealth) psychotherapeutic interventions, such as internet CBT (iCBT), treat psychological problems via digital platforms [12]. The eHealth approach involves adapting standard face-to-face protocols into computerized self-help material delivered over a period either as a self-help program alone or combined with brief therapist support [4]. Similar to face-to-face interventions, most randomized controlled trials (RCTs) in eHealth psychotherapy have evaluated interventions with CBT components [6].

Available meta-analyses suggest that internet-based psychotherapeutic interventions are effective for DDs compared to a waiting list or attention control condition. Internet-based interventions improved depression severity in adults with major depression (SMD –0.90, 95% CI –1.07 to –0.73) [13]. Self-guided iCBT was effective on depressive symptom severity (SMD 0.27) and treatment response (odds ratio 1.95, 95% CI 1.52-2.50) [14]. Internet-based interventions could also be effective for treating subthreshold depression and preventing major depression [15]. Recent meta-analyses of direct comparisons suggest similar effectiveness between internet-based and face-to-face psychotherapeutic interventions for treating DDs [6,13,16-18], which was the starting point of this project.

Although the previous data suggest that iCBT can be as effective as face-to-face CBT for treating DDs, the evidence is not conclusive. Other systematic reviews found that iCBT is more effective than face-to-face CBT at reducing symptom severity in depression (SMD –1.73, 95% CI –2.72 to –0.74) [19], but there was no evidence of an effect of iCBT compared to active treatments regarding depressive symptoms in adolescents and young adults [20]. Conversely, heterogeneity between studies was substantial in the published meta-analyses, with *I*-squared (*I*^2^) up to 98%. In addition, no moderators of treatment effects were identified [20] that explained this heterogeneity. Thus, the comparative effectiveness of eHealth and face-to-face interventions is unclear.

At present, using technology to maximize accessibility for depression treatments is an important next step [6]. Digital interventions can increase access to evidence-based psychotherapeutic interventions, which explains the large-scale investments to integrate eHealth into regular health care services [4,6,21]. This context requires understanding under what conditions eHealth interventions work, if they are effective for everyone, and in which groups the interventions might be more or less effective [22,23]. For example, there are barriers to using iCBT for patients, such as computer literacy, language, and disabilities, and patients’ attrition rates can differ between face-to-face and digital interventions [24]. Additionally, psychologists must be trained to deliver eHealth interventions [6]. Finally, recommendations for using iCBT should be underpinned by high-quality evidence from studies including complex presentations of depression—that is, patients with comorbidities, higher depression severity, or risk of suicide. In summary, the evidence on digital psychotherapeutic interventions should apply to everyday practice and all patient groups [6].

The baseline characteristics of patients in the RCTs of eHealth interventions for DDs have received little consideration. Determining whether these characteristics differ among eHealth and face-to-face intervention trials is essential to gain knowledge about the external validity of eHealth trials. The aim of our study was to compare the baseline characteristics of patients with DDs included in the RCTs of eHealth and face-to-face psychotherapeutic interventions with a cognitive component.

## Methods

### Registration

This meta-epidemiological study was prospectively registered in PROSPERO (registration CRD42019085880).

### Inclusion Criteria

#### Date

We included RCTs published as an article in any language from 1990 to November 2017.

#### Participants

Participants included adults (aged ≥16 years) with a diagnosis of DD according to an established diagnostic procedure. Depression could be the only diagnosis or coexist with other conditions, but DD should be the leading psychological diagnosis. We excluded studies with patients who are hospitalized.

#### Interventions

Psychotherapeutic interventions with at least 5 sessions and a cognitive component—that is, cognitive therapy, CBT, and interpersonal therapy—were eligible. We tried to reduce the heterogeneity among the included psychotherapeutic interventions by focusing on those with a cognitive component. From now on, we will label these interventions as CBT, since they share basic principles, such as that cognitions contribute to the maintenance of depression via their association with emotions and behaviors [25]. We excluded supportive therapy, psychodynamic treatment, and interventions delivered at group level (group, family, or couple therapies).

As a comparator, the studies should have another psychotherapeutic intervention, a sham intervention, or an inactive control (such as a waiting list). To reduce heterogeneity among the included studies, we excluded pharmacological treatment or bibliotherapy as comparators, since the motivation to participate in these trials may differ. However, we admitted antidepressants with stable dosage as cointervention, as the combination of antidepressants and psychotherapeutic interventions reflects routine practice in managing DDs. We created 2 subgroups of studies based on the following criteria.

1. eHealth CBT interventions (iCBT): This group included studies evaluating the effects of an eHealth CBT intervention (internet- or device-based self-help program delivered via computer or smartphone). The iCBT must be provided by a health professional with minimum or absent email support. We excluded studies with regular or direct web-based contact (eg, web-based session or chat) or using bibliotherapy on screen. We acknowledge that these can also be eHealth interventions, but we focused on interventions requiring patients working on their own.
2. Face-to-face CBT interventions (CBT): This group included studies evaluating the effects of a CBT intervention delivered face-to-face—that is, the sessions require the patient and therapist being in the same room with direct contact. We excluded interventions delivered without visual contact—for example, communication via chat or phone exclusively.

#### Outcomes

We compared the patients’ characteristics at baseline, as shown in Textbox 1.

**Primary and secondary outcomes.**

**Primary outcomes**

- Age (years; mean, SD)
- Gender (proportion of women)
- Education (proportion of patients with higher education; ie, at least a high school degree)
- Living area (proportion of patients living in a metropolitan area)
- Depression score (mean, SD)
- Depression duration (years; mean, SD)
- History of depression (proportion of patients with at least one previous episode of depression)
- Previous depression treatment (proportion of patients who had received any kind of treatment for depression; ie, psychotherapy, antidepressants, or both)
- Actual antidepressant medication (proportion of patients receiving antidepressants at the start of [and during] the study)
- Actual physical comorbidity (proportion of patients having at least one physical comorbidity; eg, diabetes mellitus)
- Actual mental comorbidity (proportion of patients having at least one additional mental disorder; eg, Axis I diagnosis)
- Study dropout (proportion of patients who dropped out or did not finish the study)

**Secondary outcomes**

- Quality of life (measured with a validated scale; mean, SD)
- Proficiency with computers (measured with a validated scale; mean, SD)
- Having children (proportion of patients having children)
- Family status (proportion of patients living alone; ie, single, divorced, or widowed)
- Employment (proportion of patients being employed)

### Search Methods for the Identification of Studies

First, we searched the following sources for face-to-face CBT intervention studies: (1) the database of Cuijpers et al [26] (date consulted: November 11, 2017), which was created in 2009, contains the RCTs of psychological treatments for depression, and on the date of our search (November 11, 2017), consisted of a total of 351 records; and (2) the collection of articles at the Institute for Complementary and Integrative Medicine (University Hospital Zurich and University of Zurich, Switzerland).

Second, we searched the following electronic databases (from October 16 to December 31, 2017) for iCBT studies: (1) MEDLINE (via PubMed), (2) Embase, (3) PsycINFO, and (4) Google Scholar. The search strategies combined relevant search terms related to the main concepts of the search (depression, eHealth, and RCTs). We also screened the bibliographies of key publications (see Multimedia Appendix 1).

### Selection of Studies

In the first stage, 1 author (VA) screened the records (titles and abstracts) in the database of Cuijpers et al [26] to select potentially eligible face-to-face CBT studies. VA also screened the results of the electronic searches and the bibliographies of key publications for iCBT studies that could possibly be matched with the face-to-face CBT studies. If there was any uncertainty based on the information given in the title or abstract, VA asked another author (JB) for clarification. Subsequently, 2 authors assessed the eligibility of the retrieved full texts: VA assessed the eligibility of each full text, which was cross-checked by JB. Disagreements were resolved by discussion. In the second stage, 2 researchers (VA and Lena Kümmel) independently checked if the potentially eligible studies reported all the necessary data. VA and Lena Kümmel resolved disagreements by discussion, and if there was no consensus, JB reached a final decision.

To include a study in the analysis, it must have had a matching study (being either iCBT or face-to face CBT) for all the following factors (all of them predefined and implemented in this order): (1) the same depression measurement or scale (eg, Edinburgh Postnatal Depression Scale), (2) similar depression treatment (eg*,* CBT), (3) similar diagnosis, (4) similar age range, (5) similar recruitment timeframe (less than 5 years of difference), (6) similar publication dates (less than 5 years of difference), (7) similar country, and (8) similar race. VA implemented the matching, and JB checked the decisions.

### Data Extraction

Next, 2 researchers (Lena Kümmel and VA) independently used a Microsoft Excel form to extract data on participants, interventions, comparators, outcomes, and matching criteria. JLA cross-checked the extracted numerical data. Discrepancies were resolved by discussion. We did not assess the risk of bias or contact the study authors to clarify unclear information.

For each outcome, we extracted the total number of randomized participants, the number of participants with the characteristic (dichotomous data), and the mean and SD (continuous data). If different scales were used for the same construct, we standardized each study’s mean and SD to a 100-point scale. To standardize means, we applied the following formula: *(mean – lowest scale value)* *× 100/scale range*. To standardize the SDs, we multiplied the observed SD by 100 and divided it by the scale range. As this standardization method does not correct for differences in the direction of the scales, we checked that all the scales pointed in the same direction; for example, if higher values indicated a better health state. If different time units were used, we converted the time unit to years.

For missing SDs, we first tried to calculate them from the report using the Review Manager calculator (version 5.4.1; The Cochrane Collaboration) [27]. We followed the method of Wan et al [28] to estimate the mean and SD from the sample size, median, range, and IQR. If these procedures were not possible, we borrowed the SD from other studies in the same meta-analysis [29]. If several candidate SDs were available, we used the largest SD.

We attempted to perform an “available-case analysis” of the randomized population: we took as denominators the randomized participants with a complete baseline measurement of the outcome. We considered the randomized population if the population measured at baseline was unclear. When authors presented the baseline information for those who completed the intervention and those lost to follow-up separately, we pooled the data with the Review Manager calculator (version 5.4.1; The Cochrane Collaboration) [27].

### Statistical Analysis

We used the Comprehensive Meta-Analysis software (version 3; Biostat) [30] to perform the analyses. We applied a DerSimonian and Laird random-effects meta-analysis [31]. The logit transformation was used for meta-analyses of prevalence. We investigated statistical heterogeneity in the results by considering the chi-square *P* value and the *I*² statistic [32]. When the relevant levels of heterogeneity were found, we still meta-analyzed the data.

To test whether each baseline characteristic differed in CBT and iCBT trials, we used a mixed-effects model. This model pools the studies within each subgroup using the random-effects model and tests for differences between the subgroups using a fixed-effects model [33]. Due to the low number of studies (below 30 per subgroup), we used a common among-study variance (τ^2^) for each subgroup, which was computed by pooling the among-study variances of the 2 subgroups [33]. We ran a *z* test to compare the 2 effect sizes directly. The threshold for statistical significance was .05. For continuous outcomes, we also estimated the difference in means between subgroups along with its 95% CI [33]. We did not obtain a 95% CI for the difference in prevalence as there is no meaningful way to compute it [33].

## Results

### Search Result

**Figure 1:**
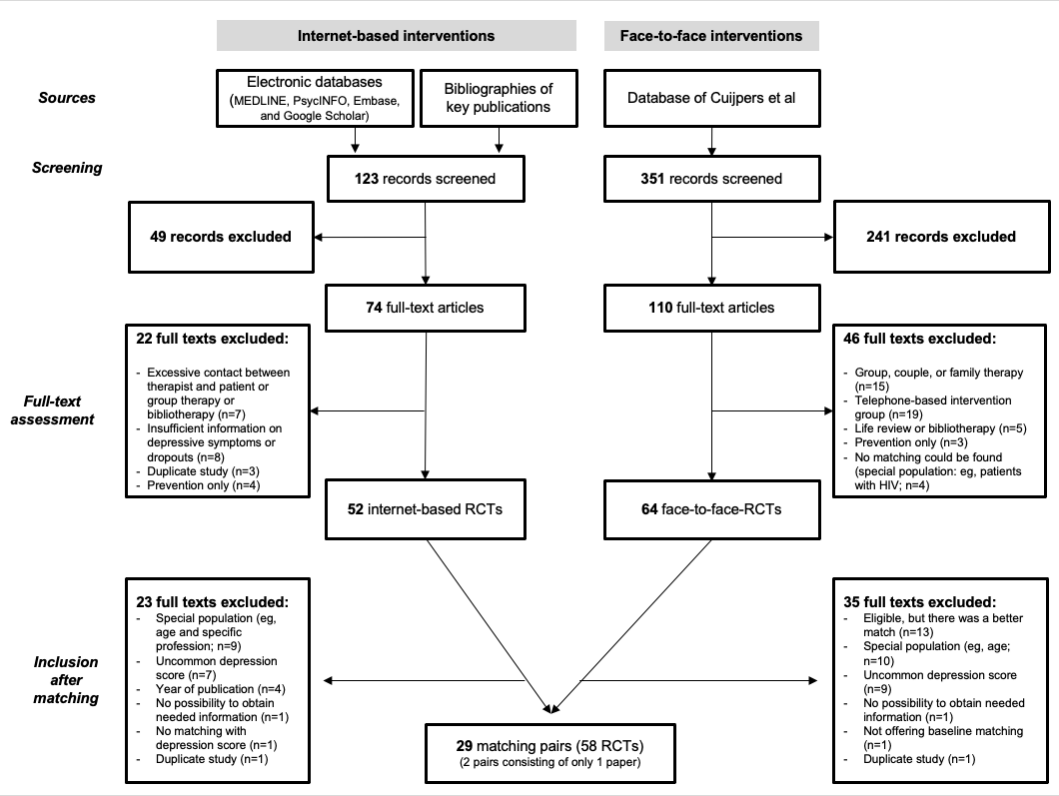
Flow chart. RCT: randomized controlled trial. Face-to-face studies screened from the database of Cuijpers et al [26].

The search for iCBT studies generated 123 records, and the search for face-to-face studies found 351 records. Therefore, we screened 474 titles and abstracts and excluded 290. We examined 184 full-text reports, of which 68 were excluded. We further assessed 64 face-to-face and 52 internet-based full-text RCTs, from which we finally included 29 matching pairs (with a total of 58 included RCTs). More details are provided in Figure 1.

### Description of the Included Studies

Multimedia Appendices 2 and 3 detail the characteristics of the 58 included studies, which were published between 1990 and 2017 (n=52, 90% of them after 2000). All studies were carried out in high-income countries: 50% (n=29) in Europe, 22% (n=13) in the United States, 17% (n=10) in Australia, and 10% (n=6) in other countries. There were no multinational studies.

The studies included a total of 3846 patients. The patients were adults (mean ages ranging from 20 to 74 years in the 57 studies reporting this information) and mostly female (n=2803, 72.9%). In the 29 studies reporting the patients’ race, White patients were the most frequently reported, representing from 63.6% to 100% of the samples. The participants’ socioeconomic status was poorly described in the 58 studies: 39 (67%) studies reported the participants’ education, ranging from college to doctoral degrees. There were 27 (47%) studies that reported the participants’ employment status: from 14% to 80% of the patients were employed (full- or part-time). There were 4 (7%) studies that reported the social class or income of the included participants: from 16% to 36% of the participants had a social class I/II or an income higher than US $30,000/year.

The included patients had different types of DDs: mild to moderate, major depression, postnatal depression, and others. Depression severity was measured with different tools, with the Beck Depression Index being the most common (n=20, 34%). The participants presented mental (eg, addictions) and physical (eg, diabetes mellitus or cardiac surgery) comorbidities that were matched in the study pairs.

All the included studies delivered psychotherapeutic interventions with a cognitive component. The duration of the interventions ranged from 6 to 20 weeks with daily, weekly, or fortnightly sessions that lasted from 10 to 90 minutes each. The cointerventions were poorly described.

### Comparison of Baseline Characteristics of Participants in the Included iCBT and Face-to-Face CBT RCTs

The results are provided in Table 1. Multimedia Appendix 4 also details the results of the meta-analyses, including the iCBT and face-to-face CBT studies.

**Table 1:** Comparison of eHealth and face-to-face psychotherapeutic studies according to baseline characteristics. No study provided data for the outcome “proficiency with computers.”

Characteristic			Meta-analyses (random-effects model)							
		Estimate (95% CI)		Study, n	Participant, n	*I*^2^ (%)	Subgroup analyses^a^			
							Difference (95% CI)^b^		*P* value	
**Age (years)**								–1.89 (–10.08 to 6.29)		.65
	iCBT^c^	39.98 (35.22-44.74)		29	2574	99.7				
	Face-to-face CBT^d^	41.81 (35.21-48.54)		28	1056	99.5				
**Gender, women, %**								—^e^		.16
	iCBT	72.9 (69.2-76.4)		29	2575	66				
	Face-to-face CBT	68.2 (62.2-73.7)		29	1271	71.1				
**Education, higher education, %**								—		.38
	iCBT	84.1 (77.8-88.8)		22	1801	86.4				
	Face-to-face CBT	79.2 (67.4-87.5)		15	789	85.2				
**Living area, metropolitan area, %**								—		.38
	iCBT	99.5 (96.2-99.9)		2	345	36.9				
	Face-to-face CBT	98.1 (88-99.7)		2	52	<0.001				
**Depression score^f^ (standardized to a 0-100–point scale)**								1.10 (–3.43 to 5.61)		.64
	iCBT	41.34 (37.37-45.31)		29	2581	98.6				
	Face-to-face CBT	42.25 (38.09-42.41)		28	1020	96.5				
**Depression duration (years)**								7.19 (2.53-11.84)		.002
	iCBT	10.0 (5.6-14.4)		1	36	0				
	Face-to-face CBT	2.8 (1.2-4.4)		5	155	89.2				
**History of depression, %**								—		.42
	iCBT	56.6 (39-72.7)		10	774	93.3				
	Face-to-face CBT	65.1 (53.1-75.5)		10	342	73.3				
**Previous depression treatment^g^, %**								—		.04
	iCBT	24.8 (18-33.1)		8	908	75.2				
	Face-to-face CBT	42 (28.3-57.1)		7	303	80.9				
**Actual antidepressant medication, %**								—		.11
	iCBT	33.1 (23.6-44.2)		13	1419	91.3				
	Face-to-face CBT	14.8 (5-36.6)		13	423	85.3				
**Actual physical comorbidity, %**								—		.33
	iCBT	99.6 (97.3-99.9)		2	254	0				
	Face-to-face CBT	98.5 (90-99.8)		2	66	0				
**Actual mental comorbidity, %**								—		.77
	iCBT	73.8 (39.2-92.5)		5	132	84.6				
	Face-to-face CBT	66.9 (28.7-91.1)		5	196	89.7				
**Study dropout, %**								—		.36
	iCBT	19.5 (14.1-26.4)		24	1878	89.5				
	Face-to-face CBT	15.4 (10.1-22.7)		24	987	83.4				
**Quality of life (standardized to a 0-100–point scale)**								14.50 (–12.54 to 41.53)		.29
	iCBT	48.11 (36.5-59.62)		9	904	99.3				
	Face-to-face CBT	33.61 (9.15-58.07)		2	90	98.5				
**Having children, %**								—		.55
	iCBT	99 (95.3-99.8)		3	221	0				
	Face-to-face CBT	98.1 (91-99.6)		3	79	0				
**Family status, living alone, %**								—		.37
	iCBT	38.3 (30.8-46.5)		20	1795	88.2				
	Face-to-face CBT	44.2 (34.5-54.4)		20	768	83.3				
**Employment, %**								—		.45
	iCBT	59.4 (47.9-69.9)		13	1413	90.6				
	Face-to-face CBT	53 (40.9-64.8)		13	519	80.9				

^a^Degrees of freedom=1.

^b^95% CI for the difference in prevalence was not calculated, as there is no meaningful way to compute it.

^c^iCBT: internet cognitive behavioral therapy.

^d^CBT: cognitive behavioral therapy.

^e^Not available.

^f^Subgroup analyses for depression measured with individual scores: Beck Depression Inventory (*P≥*.99); Depression, Anxiety and Stress Scale-21 (*P*=.04); Center for Epidemiologic Studies-Depression Scale (6 studies; *P*=.87), Edinburgh Postnatal Depression Scale (4 studies; *P*=.34), and Hamilton Depression Rating Scale (6 studies; *P*=.07).

^g^Proportion of patients (%) having received any kind of treatment for depression (ie, psychotherapy, antidepressants, or both).

#### Primary Outcomes

The mean depression duration was 7.19 years higher (CI 95% 2.53-11.84) in iCBT trials than in face-to-face CBT trials (10.0 vs 2.8 years; *P*=.002). However, the mean proportion of patients with previous depression treatment was lower in iCBT trials (24.8% vs 42% in face-to-face trials; *P*=.04). The subgroup analyses found no evidence of differences for the remaining primary outcomes: age, gender, education, living area, depression score, history of depression, actual antidepressant medication, actual physical comorbidity, actual mental comorbidity, and study dropout.

#### Secondary Outcomes

We found no evidence of differences between iCBT and face-to-face CBT studies for quality of life, having children, family status, and employment. Subgroup analysis for the proficiency with computers could not be performed due to insufficient studies.

## Discussion

### Principal Findings

To our knowledge, our study is the first to compare the baseline characteristics of patients with DDs included in the RCTs of eHealth and face-to-face CBT interventions. Overall, we found that the patients’ characteristics between eHealth and face-to-face RCTs were generally similar. This finding suggests that patients in both types of trials are comparable rather than different. However, patients in eHealth trials had a longer depression duration, and a lower proportion had received previous depression treatment.

eHealth psychological interventions have several advantages compared to face-to-face interventions. First, iCBT creates the opportunity to deliver psychological treatment to people without access to face-to-face therapy [12]. Thus, iCBT can help patients avoid traveling to physical consultations and can mitigate the shortage of professionals [6,34-36]. Second, guided iCBT probably represents the most economical option for the short-term treatment of adults with mild-to-moderate major depression [24]. Third, eHealth interventions have become highly automated, which enhances fidelity with treatment protocols [33,37]. Fourth, digital interventions are becoming acceptable for patients and therapists, particularly since the COVID-19 pandemic [6,24,38]. In fact, surveys suggest that iCBT guided by therapists could become a preferred option for patients over in-person CBT or medication [39].

We assumed that the baseline characteristics of patients in eHealth and face-to-face psychotherapeutic intervention RCTs would differ. For example, we hypothesized that patients in eHealth RCTs would be younger due to their familiarity with computers and frequent use of social media [40]. However, we did not find differences regarding the patients’ age, which is supported by recent literature that suggests that older adults are becoming more computer literate and that iCBT could therefore be a treatment opportunity [41]. Conversely, available research suggests that younger people are more likely to drop out of iCBT, but the reason for that result remains unknown [42]. Our study was not able to confirm this finding.

Our study found that patients in eHealth RCTs presented a longer depression duration but had received previous depression treatment in a lower number. The longer depression duration in eHealth RCTs could be explained by the fact that patients in eHealth trials perceive barriers concerning face-to-face treatments, and therefore, eHealth treatment might be more attractive to them. Conversely, we expected that patients in eHealth RCTs would present more severe depression since a lower proportion had received treatment for depression. However, our analyses did not support this assumption. Finally, our findings might indicate that eHealth trials attract patients who postpone earlier treatment attempts, but future research should be conducted to confirm this finding.

Our searches identified a high number of RCTs, which confirms the recent expansion of research into digital interventions [6,43]. However, the baseline characteristics of the trial participants were poorly and heterogeneously reported. This finding represents a major limitation of the available research, as the role of these characteristics is essential to understand under what conditions eHealth psychotherapeutic interventions will work. For example, few studies reported the patients’ proficiency with computers, which is critical to explain the lack of effect of a digital intervention. Additionally, there is room for improvement in the reporting of socioeconomic characteristics, such as working conditions or family status, which are essential features to understand which type of patients benefit from eHealth interventions. Furthermore, it would be of interest to know about the comorbidities directing a patient to a certain treatment method as well as previous treatments against DDs.

This incomplete reporting highlights the need to agree to a consensus-based minimum set of baseline characteristics that should be measured and reported in all RCTs of eHealth psychotherapeutic interventions. Once the list is defined, consensus should be achieved on how to measure these characteristics, such as which measurement instruments should be selected to measure proficiency with computers. Finally, the reporting of these characteristics should be encouraged in future RCTs to allow the assessment of the applicability of the study findings.

### Strengths and Limitations

Our study had several limitations, but we tried to overcome them by following rigorous methods [44].

First, our searches may have missed eligible studies. Particularly, we limited the searches from 1990 onward as no eligible study would have been published before. The restriction until 2017 was because the searches were executed that year, and we did not have the resources to update them. However, we did not attempt to perform a systematic review and, thus, include all the studies in this field. We consider that the 58 included RCTs probably give an unbiased view of the situation in this research field.

Second, our matching process by relevant characteristics may have minimized differences between subgroups. Moreover, otherwise eligible studies were excluded because we could not find their matching pair. However, we consider that the matching process minimized confounding in the subgroup analyses (see below).

Third, subgroup analysis is a technique with considerable pitfalls. Nevertheless, we followed established guidelines to overcome the main limitations. (1) We prevented post hoc analyses and undue emphasis on particular findings by choosing the analyses in advance with clear rationale [45,46]. (2) We found a high number of studies for most outcomes, which increased the statistical power (which is usually low in subgroup analyses). For example, 6 analyses presented at least 20 studies per subgroup. (3) We did not simply compare the statistical significance of the results in each subgroup and performed formal significance tests [31,33]. Moreover, we estimated the difference in means between subgroups and its 95% CI for continuous outcomes, which allowed us to judge if the differences were clinically relevant [33]. (4) We interpreted the results cautiously. We acknowledge that subgroup comparisons are exploratory analyses that are observational by nature [31,44]. As studies are not allocated randomly to each subgroup, we cannot assume that the subgroup populations were identical except for the intervention type (internet-based or face-to-face). Thus, the results from a subgroup analysis are prone to confounding by other study-level characteristics [31]. Similarly, although we found differences in 2 characteristics between subgroups (depression duration and previous treatment for depression), we cannot conclude that they were due to the type of intervention delivery (eHealth or face-to-face).

Fourth, there is an increasing risk of type 2 error concurrent with the number of analyses, which was 16 in our case [31]. Although we did not adjust the significance level to account for multiple analyses, we assessed the impact of this decision by sensitivity analysis. After setting a new .006 threshold for statistical significance according to the proposal by Jakobsen et al [47], only the depression duration at baseline maintained its statistical significance. Thus, our general conclusion did not change; overall, patients in iCBT and face-to-face RCTs had similar sociodemographic and depression characteristics.

### Conclusions

This is the first study comparing the baseline characteristics of patients with DDs included in the RCTs of eHealth and face-to-face psychotherapeutic interventions. Overall, our study did not find differences in the patients’ characteristics between eHealth and face-to-face RCTs. However, patients in eHealth trials had a longer depression duration, and a lower proportion had received previous depression treatment. This finding might indicate that eHealth trials attract patients who postpone earlier treatment attempts. Our findings highlight a need to improve the reporting of the baseline characteristics of patients included in the RCTs of eHealth psychotherapeutic interventions.

## Appendix

Multimedia Appendix 1 Key publications.

Multimedia Appendix 2 Included studies.

Multimedia Appendix 3 Summary of the included studies.

Multimedia Appendix 4 Meta-analyses of baseline characteristics.

## Abbreviations

CBT: cognitive behavioral therapy
DD: depressive disorder
iCBT: internet cognitive behavioral therapy
RCT: randomized controlled trial
SMD: standardized mean difference
