# Supplementary material for: Comparability of Patients in Trials of eHealth and Face-to-Face Psychotherapeutic Interventions for Depression: Meta-synthesis
Source: J Med Internet Res. 2022 Sep 14;24(9):e36978. doi: 10.2196/36978 (PMC9520399; doi:10.2196/36978)
Supplement: Multimedia Appendix 1 [file jmir_v24i9e36978_app1.docx]

**Appendix 1. Key Publications**

We screened the bibliographies of the following publications

Andersson, G. (2009). Using the Internet to provide cognitive behaviour therapy. *Behaviour research and therapy, 47*(3), 175-180. doi:10.1016/j.brat.2009.01.010

Andersson, G., & Cuijpers, P. (2009). Internet-based and other computerized psychological treatments for adult depression: a meta-analysis. *Cognitive Behaviour Therapy, 38*(4), 196-205. doi:10.1080/16506070903318960

Andersson, G., Cuijpers, P., Carlbring, P., Riper, H., & Hedman, E. J. W. P. (2014). Guided Internet‐based vs. face‐to‐face cognitive behavior therapy for psychiatric and somatic disorders: a systematic review and meta‐analysis. *World Psychiatry, 13*(3), 288-295.

Andrews, G., Cuijpers, P., Craske, M. G., McEvoy, P., & Titov, N. (2010). Computer therapy for the anxiety and depressive disorders is effective, acceptable and practical health care: a meta-analysis. *PLoS One, 5*(10), e13196. doi:10.1371/journal.pone.0013196

Barth, J., Michlig, N., & Munder, T. (2014). Unique and shared techniques in cognitive-behavioural and short-term psychodynamic psychotherapy: a content analysis of randomised trials on depression. *Health Psychology and Behavioral Medicine, 2*(1), 929-950. doi:10.1080/21642850.2014.931231

Barth, J., Munder, T., Gerger, H., Nuesch, E., Trelle, S., Znoj, H., . . . Cuijpers, P. (2013). Comparative efficacy of seven psychotherapeutic interventions for patients with depression: a network meta-analysis. *PLoS Medicine, 10*(5), e1001454. doi:10.1371/journal.pmed.1001454

Carlbring, P., Andersson, G., Cuijpers, P., Riper, H., & Hedman-Lagerlof, E. (2018). Internet-based vs. face-to-face cognitive behavior therapy for psychiatric and somatic disorders: an updated systematic review and meta-analysis. *Cognitive Behaviour Therapy, 47*(1), 1-18. doi:10.1080/16506073.2017.1401115

Cuijpers, P., Donker, T., Johansson, R., Mohr, D. C., van Straten, A., & Andersson, G. (2011). Self-guided psychological treatment for depressive symptoms: a meta-analysis. *PLoS One, 6*(6), e21274. doi:10.1371/journal.pone.0021274

Cuijpers, P., Donker, T., van Straten, A., Li, J., & Andersson, G. (2010). Is guided self-help as effective as face-to-face psychotherapy for depression and anxiety disorders? A systematic review and meta-analysis of comparative outcome studies. *Psychological medicine, 40*(12), 1943-1957. doi:10.1017/S0033291710000772

Cuijpers, P., van Straten, A., Warmerdam, L., & Andersson, G. (2008). Psychological treatment of depression: a meta-analytic database of randomized studies. *BMC Psychiatry, 8*, 36. doi:10.1186/1471-244X-8-36

Fernandez, E., Salem, D., Swift, J. K., & Ramtahal, N. (2015). Meta-analysis of dropout from cognitive behavioral therapy: magnitude, timing, and moderators. *Journal of Consulting and Clinical Psychology, 83*(6), 1108-1122. doi:10.1037/ccp0000044

Johansson, R., & Andersson, G. (2012). Internet-based psychological treatments for depression. *Expert review of neurotherapeutics, 12*(7), 861-869; quiz 870. doi:10.1586/ern.12.63

Karyotaki, E., Kleiboer, A., Smit, F., Turner, D. T., Pastor, A. M., Andersson, G., . . . Cuijpers, P. (2015). Predictors of treatment dropout in self-guided web-based interventions for depression: an 'individual patient data' meta-analysis. *Psychological medicine, 45*(13), 2717-2726. doi:10.1017/S0033291715000665

Lindner, P., Nyström, M. B. T., Hassmén, P., Andersson, G., & Carlbring, P. (2015). Who seeks ICBT for depression and how do they get there? Effects of recruitment source on patient demographics and clinical characteristics. *Internet Interventions, 2*(2), 221-225. doi:10.1016/j.invent.2015.04.002

Richards, D., & Richardson, T. (2012). Computer-based psychological treatments for depression: a systematic review and meta-analysis. *Clinical psychology review, 32*(4), 329-342. doi:10.1016/j.cpr.2012.02.004

Zagorscak, P., Heinrich, M., Sommer, D., Wagner, B., & Knaevelsrud, C. (2018). Benefits of individualized feedback in internet-based interventions for depression: a randomized controlled trial. *Psychotherapy and Psychosomatics, 87*(1), 32-45. doi:10.1159/000481515
