# Supplementary material for: Comparability of Patients in Trials of eHealth and Face-to-Face Psychotherapeutic Interventions for Depression: Meta-synthesis
Source: J Med Internet Res. 2022 Sep 14;24(9):e36978. doi: 10.2196/36978 (PMC9520399; doi:10.2196/36978)
Supplement: Multimedia Appendix 2 [file jmir_v24i9e36978_app2.docx]

| **Appendix 2. Included studies, listed in matching pairs** | | | | | |
| --- | --- | --- | --- | --- | --- |
| Setting  First Author (Publication year) | Reference | ● Diagnosis  ● Depression measure | ● Participants’ age (years)  ● Recruitment method  ● Study recruitment dates  ● Country | Total study sample // Intervention group sample size | ● Intervention  ● Provider  ● Intervention (total nr. of sessions, duration of a session, periodicity, and total duration)  ● Setting  ● Co-interventions |
|  |  |  |  |  |  |
| ftf  Milgrom (2011) | Milgrom, et al. Treating postnatal depressive symptoms in primary care: a randomised controlled trial of GP management, with and without adjunctive counselling. BMC psychiatry. 2011;11(1):95 | ● Postnatal depression  ● EPDS | ● Mean: 31.4 years (SD NR)  ● Postnatal women (infants < 12 months of age) were screened by nurses working in primary care at maternal child health centers during regular routine visits  ● Study recruitment dates: NR  ● Country: Australia | 68 // 23 | ● Counselling-CBT  ● Experienced psychologist  ● 6 sessions (duration NR), weekly, 6 weeks in total  ● Primary care setting (general practices and maternal & child health centers), a psychology clinic  ● GP management |
| iCBT  O'Mahen (2013) | O'Mahen HA, et al. Internet-based behavioral activation-treatment for postnatal depression (Netmums): a randomized controlled trial. Journal of affective disorders. 2013;150(3):814-22 | ● Postnatal depression  ● EPDS | ● Mean: 32.3 years (SD 4.7)  ● Via a popular UK parenting site (netmums.com)  ● Study recruitment dates: 09/2009-01/2010  ● Country: UK | 910 // 172 | ● Postnatal, online delivery from a manual developed for BA, adapted for the postnatal period  ● A specialist health visitor or a clinical psychologist  ● 11 sessions (up to 40 min per session), number per week NR, maximal 15 weeks in total  ● At home  ● None |
|  |  |  |  |  |  |
| ftf  Power (2012) | Power MJ, Freeman C. A randomized controlled trial of IPT versus CBT in primary care: with some cautionary notes about handling missing values in clinical trials. Clinical Psychology & Psychotherapy. 2012;19(2):159-69. | ● SCID diagnosis of depression  ● BDI | ● Mean: 36.1 years (SD 11.3)  ● Referred by GPs working in Primary Care both in Edinburgh and the surrounding areas in Lothian  ● Study recruitment dates: NR  ● Country: UK | 125 // 19 | ● CBT arm (followed the Beck et al. manual, (2))  ● 9 clinical psychologists, 2 psychiatrists and 5 nurse therapists  ● 12 - 16 sessions, duration, periodicity, and total duration NR  ● NR  ● None |
| iCBT  Andersson (2013) | Andersson G, et al. Randomised controlled non-inferiority trial with 3-year follow-up of internet-delivered versus face-to-face group cognitive behavioural therapy for depression. Journal of affective disorders. 2013;151(3):986-94 | ● Mild to moderate depression  ● BDI | ● Mean: 42.8 years (SD 14.9)  ● Recruited by means of articles in regional newspapers, posters at the University campus, local health care facilities, and information on web pages  ● Study recruitment dates: NR  ● Country: Sweden | 69 // 33 | ● Guided internet CBT (self-help treatment); patients were instructed to submit homework assignments each Sunday after which they received personalized feedback within 24h  ● 4 students of a psychology program (5-year Msc)  ● 7 text modules (e.g., chapters) with 114 pages in total (duration NR), approximately 1 module per week, total duration NR  ● At home  ● None |
|  |  |  |  |  |  |
| ftf  David (2008) | David D, et al. Rational emotive behavior therapy, cognitive therapy, and medication in the treatment of major depressive disorder: a randomized clinical trial, posttreatment outcomes, and six-month follow-up. Journal of clinical psychology. 2008;64(6):728-46 | ● Nonpsychotic major depressive disorder (DSM-IV)  ● BDI | ● Mean: 39 years (SD 10)  ● Directly referred from several psychotherapy centers, University, and private practice clinics.  ● Study recruitment dates: 2001-2004  ● Country: Romania | 170 // 56 | ● CT treatment (following a manual by the author) including behavioral activation, dysfunctional thoughts modification, the identification and structural modification of generalized core beliefs  ● 8 experienced therapists (6 psychologists, 2 psychiatrists)  ● 20 sessions (50-minutes), periodicity NR, total duration of 14 weeks  ● NR  ● None |
| iCBT  Berger (2011) | Berger T, et al. Internet-based treatment of depression: a randomized controlled trial comparing guided with unguided self-help. Cognitive behaviour therapy. 2011;40(4):251-66 | ● Major depression or dysthymia according to DSM-IV  ● BDI | ● Mean: 38.6 years (SD 14.2)  ● Articles in national and regional newspapers, a national television interview or via web page.  ● Study recruitment dates: NR  ● Country: Switzerland and Germany | 76 // 25 | ● “Deprexis”^1^ program: unguided web-based self-help treatment; without any support from a therapist  ● 2 female Master students of clinical psychology and psychotherapy Master, 1 female psychologist, 1 male CBT therapist with research and clinical experience  ● 10 content modules with a summary module covering a variety of therapeutic content (broadly consistent with a cognitive behavioral model), 10–60 minutes per module, periodicity NR, 10 weeks in total  ● NR  ● None |
|  |  |  |  |  |  |
| ftf  Levin (2011) | Levin W, et al. A computer-assisted depression intervention in primary care. Psychological medicine. 2011;41(7):1373-83 | ● DSM-IV criteria for depressed mood or anhedonia  ● CES-D | ● Mean: 44 years (SD 13.3)  ● Through primary-care physicians (PCPs) in Lane County (Oregon), identified from medical directory listings and the local phone book  ● Study recruitment dates: NR  ● Country: USA | 191 // 100 | ● “WW (Wellness Workshop)”, a National Institutes of Health-supported, stand-alone interactive instructional program on CD-ROM for delivery on a personal computer  ● Therapists had experience in a mental health field and complete a rigorous training program in the use of the SCID  ● Number of sessions, duration, periodicity and weeks in total NR; active participants used the program for an average of 2.9 weeks (SD=1.8, range=1–8)  ● Computer sites at hospital-based health education, libraries or at home  ● TAU with GP |
| iCBT  Buntrock (2015) | Buntrock C, et al. Effectiveness of a web-based cognitive behavioural intervention for subthreshold depression: pragmatic randomised controlled trial. Psychotherapy and psychosomatics. 2015;84(6):348-58 | ● Subthreshold depression  ● CES-D | ● Mean: 45.71 years (SD 11.93)  ● From the general population via a large German health insurance company, through newspaper articles, on-air media, and related websites  ● Study recruitment dates: 03/2013 - 03/2014  ● Country: Germany | 406 // 202 | ● Web-based intervention; sessions include text, exercises, testimonials, and interactive elements such as audio and video clips  ● NR, interview done by trainees in psychotherapy  ● 6 interactive sessions (30-60 minutes), 1-2 per week, 3-6 weeks in total  ● At home  ● CAU by GP |
|  |  |  |  |  |  |
| ftf  Dekker (2008) | Dekker JJ, et al. Speed of action: the relative efficacy of short psychodynamic supportive psychotherapy and pharmacotherapy in the first 8 weeks of a treatment algorithm for depression. Journal of Affective Disorders. 2008;109(1-2):183-8. | ● DSM-IV-defined Depressive Episode with or without dysthymia  ● CGI; HAMD | ● Mean and SD NR; Range: 20 - 60 years  ● Newly registered during 3 years at two outpatient clinics of Jellinek Mentrum Mental Health Care (Amsterdam)  ● Study recruitment dates: NR  ● Country: Netherlands | 103 // 59 | ● SPSP, a manual-based approach focusing on the affective, behavioral, and cognitive aspects of relationships  ● Trained psychiatrists or psychotherapists  ● 16 sessions (duration NR), weekly sessions during the first 8 weeks, fortnightly in the second series of 8 weeks, total duration NR  ● NR  ● None |
| iCBT  Johansson (2012a) | Johansson R, et al. Psychodynamic guided self-help for adult depression through the internet: a randomised controlled trial. PLoS one. 2012;7(5):e38021. | ● MDD; according to the DSM-IV, with current acute episode of depression or an episode in partial remission  ● CGI (MADRS-S) | ● Mean: 45.5 years (SD 15.2)  ● Advertisement in a major Swedish newspaper, additional participants from a waiting list for another treatment trial for depression  ● Study recruitment dates: NR  ● Country: Sweden | 92 // 46 | ● Psychodynamic treatment (guided self-help, with minimal text-based guidance)  ● 6 final-semester students from a five-year M.Sc. clinical psychologist program  ● 9 treatment modules, totaling 167 pages of text, duration and periodicity NR, 10 weeks in total  ● Home (assumed by the reviewer)  ● None |
|  |  |  |  |  |  |
| ftf  Watkins (2012) | Watkins, E, et al. Guided self-help concreteness training as an intervention for major depression in primary care: A Phase II randomized controlled trial. *Psychological Medicine,* *42*(7), 1359-1371. | ● Current major depression or subthreshold (DSM-IV)  ● BDI (mild to moderate depression, also HAMD score); cut-off NR | ● Mean: 46.37 years (SD 12.71)  ● Computerized databases in 15 primary care practices serving a population of around 300 000 people across Devon were searched to identify patients who had been prescribed antidepressant medication or recorded as depressed on Read codes for the previous 6 months  ● Study recruitment dates: NR  ● Country: UK | 121 // 40 | ● CNT, involves patients’ identifying a recent mildly to moderately upsetting difficulty and working through standardized steps to facilitate concrete thinking  ● 2 graduate-level psychologists, 2 postdoctoral psychologists and 1 clinical psychologist  ● Patients recorded on audiotape/compact disc (CD), supported by a detailed workbook (recommended frequency of 15–30 min daily), periodicity NR, at least 6 weeks (weeks in total NR)  ● NR  ● TAU by GP |
| iCBT  de Graaf (2009) | de Graaf LE, et al. Clinical effectiveness of online computerised cognitive-behavioural therapy without support for depression in primary care: randomised trial. The British Journal of Psychiatry. 2009;195(1):73-80. | ● Mild to moderate depressive complaints  ● BDI–II | ● Mean: 44.3 years (SD 11.8)  ● A large-scale internet-based screening in the south of The Netherlands  ● Study recruitment dates: NR  ● Country: Netherlands | 303 // 100 | ● CBT program, named Colour Your Life (online, multimedia, interactive computer program for depression, based on the principles of CBT and on the Dutch version of ‘The Coping with Depression Course’^2^)  ● NR  ● 8 sessions (30-min long) and a ninth booster session; weekly, 8 weeks in total  ● NR, at home (assumed by the reviewer)  ● TAU by GP |
|  |  |  |  |  |  |
| ftf  Lexis (2010) | Lexis MAS, et al. Prevention of long-term sickness absence and major depression in high-risk employees: a randomised controlled trial. Occupational and Environmental Medicine. 2010;68(6):400-7. | ● Mild to severe depressive complaints, still at work  ● BDI-II | ● Mean: 48.41 years (SD 8.68)  ● Employees of a large banking company in The Netherlands received a screening questionnaire at their home address  ● Study recruitment dates: 2007  ● Country: Netherlands | 139 // 69 | ● PST; through all sessions, principles of CBT were applied.  ● Psychologists  ● 7-12 sessions (45 min each), periodicity and total duration NR  ● NR  ● TAU by GP or another specialist |
| iCBT  Geraedts (2014) | Geraedts AS, et al. Short-term effects of a web-based guided self-help intervention for employees with depressive symptoms: randomized controlled trial. Journal of medical Internet research. 2014;16(5):e121. | ● Elevated depressive symptoms  ● CES-D | ● Mean: 43 years (SD 8.9)  ● In 6 different (international) companies in the Netherlands: banking, research institutes, security company, and university, via banners and digital pamphlets on the companies’ intranet or via posters  ● Study recruitment dates: NR  ● Country: Netherlands | 231 // 116 | ● “Happy@Work” is a brief Web-based intervention delivered with minimal guidance containing PST and a guideline for employees to help them to prevent work-related stress.  ● Coaches: master’s students in clinical psychology  ● 6 weekly lessons (duration NR), weekly, 6-7 weeks in total  ● NR  ● NR |
|  |  |  |  |  |  |
| ftf  Kay-Lambkin (2011) | Kay‐Lambkin FJ, et al. Clinician‐assisted computerised versus therapist‐delivered treatment for depressive and addictive disorders: a randomised controlled trial. Medical Journal of Australia. 2011;195:S44-S50. | ● Depressive and addictive disorders  ● BDI-II | ● Mean: 40 years (SD NR); Range 17–70 years  ● Self-referral in response to advertisements promoting the study, public AOD outpatient treatment facilities, general practices, non-government support agencies, government-funded employment services, and public mental health outpatient clinics  ● Study recruitment dates: 01/2005 - 08/2007  ● Country: Australia | 274 // 88 | ● PCT consisting of supportive counselling  ● Intern, registered or clinical psychologists  ● 9 sessions (60-minute sessions), weekly, 9 weeks in total  ● NR  ● NR |
| iCBT  Kay-Lambkin (2009) | Kay‐Lambkin FJ, et al. Computer‐based psychological treatment for comorbid depression and problematic alcohol and/or cannabis use: a randomized controlled trial of clinical efficacy. Addiction. 2009;104(3):378-88 | ● Comorbid depression and alcohol/cannabis use problems; life-time diagnosis of major depressive disorder (DSM-IV (SCID-RV))  ● BDI-II | ● Mean: 35.37 years (SD NR); Range 18–61 years  ● AOD, mental health and primary health-care settings, from the general community, in response to advertising through the local television and print media  ● Study recruitment dates: NR  ● Country: Australia | 97 // 32 | ● Computer-delivered SHADE therapy (incorporated MI and CBT Components) including interactive components such as video demonstrations, voice-overs, and in-session exercises  ● Research clinician  ● 9 sessions (duration NR), weekly, 9 weeks in total  ● Research center to access the computer program  ● NR |
|  |  |  |  |  |  |
| ftf  Ekers (2018) | Ekers D, et al. Behavioural activation delivered by the non-specialist: phase II randomised controlled trial. British Journal of Psychiatry. 2018;198(1):66-72 | ● ICD-diagnosis of depression  ● BDI | ● Mean: 46.43 years (SD NR); Range 24–63 years  ● General practices (based in a mix of rural and urban settings) directly or from primary care mental health services over a 9-month period  ● Study recruitment dates: NR  ● Country: UK | 47 // 23 | ● BA as a step-three intervention, based on two behavioral approaches developed in previous research  ● 2 qualified mental health nurses  ● 12 face-to-face sessions (60-min), periodicity NR, 12 weeks in total  ● NR  ● NR |
| iCBT  Montero-Marin (2016) | Montero-Marin J, et al. An Internet-Based Intervention for Depression in Primary Care in Spain: A Randomized Controlled Trial. Journal of medical Internet research. 2016;18(8):e231. | ● Major depression; with mild or moderate severity symptoms according to (BDI-II)  ● BDI | ● Mean and SD NR; Range: 18-65 years  ● GPs in Spain identified potential participants through using a case-finding questionnaire  ● Study recruitment dates: 11/2012 - 01/2014  ● Country: Spain | 296 // 98 | ● CBT; Smiling is Fun as an Internet-delivered, self-help program, covering different psychological techniques for coping with depression  ● 4 trained psychotherapists (only for technical question)  ● 10 CBT-modules, duration and periodicity NR, 10 weeks in total  ● At home  ● TAU by GP |
|  |  |  |  |  |  |
| ftf  Serfaty (2009) | Serfaty MA, et al. Clinical effectiveness of individual cognitive behavioral therapy for depressed older people in primary care: a randomized controlled trial. Archives of general psychiatry. 2009;66(12):1332-40. | ● Depressive disorder (AGECAT)  ● BDI | ● Mean: 74.4 years (SD 7.6)  ● Self-referral, general practice referral (either from a direct approach by researchers or via the GP), and by database searches  ● Study recruitment dates: 04/2004 - 09/2007  ● Country: UK | 204 // 70 | ● CBT-techniques (modified for use in older people); increased structure of the sessions and techniques to facilitate recall, specific issues associated with old age were addressed with exploration of patients’ beliefs about negative effects of physical ill health and their perceptions of themselves in the context of their age  ● Accredited therapists for Behavioral and Cognitive Psychotherapies with at least 5 years practice in CBT  ● At least 6-8 sessions (up to 12 sessions) (50-minutes), weekly, duration in total NR  ● At institute or at home (if participant was unable to travel)  ● NR |
| iCBT  Spek (2007) | Spek V, et al. Internet-based cognitive behavioural therapy for subthreshold depression in people over 50 years old: a randomized controlled clinical trial. Psychological medicine. 2007;37(12):1797-806. | ● Subthreshold depression  ● BDI | ● Mean: 55 years (SD 4.9)  ● Advertisements in free regional newspapers, and by personal letters sent by the Municipal Health Care  Service; letters (n=15 697) were sent in cohorts to all inhabitants of Eindhoven, born between 1955 and 1949  ● Study recruitment dates: NR  ● Country: Netherlands | 301 // 102 | ● Internet-based CBT intervention; self-help intervention with text, exercises, videos, and figures, based on the Coping with Depression course^2^, no professional support was offered to the participants of this study  ● Psychologists and trained social workers  ● 8 modules (duration NR), weekly, 8 weeks in total  ● At home  ● NR |
|  |  |  |  |  |  |
| ftf  Chan (2012) | Chan AS, et al. A chinese chan-based mind-body intervention for patients with depression. Journal of Affective Disorders. 2012;142(1-3):283-9. | ● Major depressive disorder // DSM-IV  ● BDI | ● Mean: 46.94 years (SD 6.54)  ● Outpatients recruited from the West Kowloon Psychiatric Center  ● Study recruitment dates: NR  ● Country: China | 75 // 17 | ● CBT-protocol derived from a combination of sources, including CT for Depression, Mind Over Mood, and CBT in Groups. Typical treatment components include: Progressive Muscular Relaxation; behavioral activation; self-monitoring; cognitive restructuring, including evaluating and reconsidering interpretive and predictive cognitions; cognitive techniques for deeply held core beliefs and deep-rooted conditional assumptions; rehearsal of coping skills; and relapse prevention.  ● Clinical psychologist with over 10 years of experience  ● 10 sessions (90 minutes), weekly, total duration NR  ● NR  ● None |
| iCBT  Choi (2012) | Choi I, et al. Culturally attuned Internet treatment for depression amongst Chinese Australians: a randomised controlled trial. Journal of affective disorders. 2012;136(3):459-68. | ● DSM-IV criteria for a major depressive episode using the Chinese-Bilingual Structured Diagnostic Interview Schedule  ● CBDI | ● Mean: 40.6 years (SD 10.3)  ● Online after reading details about the study and about inclusion and exclusion criteria in the mass media  ● Study recruitment dates: NR  ● Country: China | 55 // 28 | ● Chinese depression iCBT program (The Brighten Your Mood Program) is a modified version of the clinically efficacious Sadness iCBT Program  ● Clinical psychologist  ● 6 lessons (duration NR), weekly, max of 8 weeks in total  ● At home  ● NR |
|  |  |  |  |  |  |
| ftf  Barber (2012) | Barber JP, et al. Short-term dynamic psychotherapy versus pharmacotherapy for major depressive disorder. The Journal of Clinical Psychiatry. 2012;73(01):66-73. | ● Major depressive disorder (DSM-IV)  ● BDI | ● Mean: 36.2 years (SD 12.2)  ● Through advertisements on public transportation and in free news publications, area physicians, and outpatient clinics  ● Study recruitment dates: 11/2001 - 06/2007  ● Country: USA | 156 // 51 | ● SET  ● 4 psychologists with over 15 years of experience  ● 19 sessions (45-minute sessions), twice weekly during the first 4 weeks of treatment and weekly for weeks 5-16, 16 weeks in total  ● NR  ● NR |
| iCBT  Johansson (2012b) | Johansson R, Sjoberg E, et al. Tailored vs. standardized internet-based cognitive behavior therapy for depression and comorbid symptoms: a randomized controlled trial. PLoS one. 2012;7(5):e36905. | ● Major depressive disorder DSM-IV, with a current acute episode of depression or an episode in partial remission  ● BDI | ● Mean: 45.7 years (SD 10.9)  ● Online waiting list for people interested in Internet-based treatment for depression and by an advertisement made in a large Swedish newspaper the week before the study formally started  ● Study recruitment dates: NR  ● Country: Sweden | 121 // 39 | ● The tailored treatment consisted of material on depression, panic, social anxiety, worrying and additional material e.g. on stress management, concentration problems, problem solving strategies, mindfulness, and relaxation  ● 7 MSc-level clinical psychologist students who had received clinical training  ● 25 chapters (duration NR), periodicity NR, 10 weeks in total  ● At home  ● NR |
|  |  |  |  |  |  |
| ftf  Laidlaw (2008) | Laidlaw K, et al. A randomised controlled trial of cognitive behaviour therapy vs treatment as usual in the treatment of mild to moderate late life depression. International Journal of Geriatric Psychiatry. 2008;23(8):843-50. | ● Mild to moderate Major Depressive Episode; primary diagnosis of Major Depressive Disorder using DSM IV diagnostic criteria  ● BDI | ● Mean: 74 years (SD 8.39)  ● Recruited from Primary Care and referred to the study by their GP  ● Study recruitment dates: NR  ● Country: UK | 43 // 20 | ● CBT for late life depression is an active, directive time-limited and structured problem-solving treatment approach whose primary aim is symptom reduction  ● Masters level trained Chartered Clinical Psychologists, 1 graduate psychologist  ● 8 sessions; duration, periodicity, and total duration NR  ● NR  ● None |
| iCBT  Preschl (2012) | Preschl B, et al. Life-review therapy with computer supplements for depression in the elderly: a randomized controlled trial. Aging & mental health. 2012;16(8):964-74. | ● Minimal (subsyndromal) to moderate depression  ● BDI-II | ● Mean: 72.5 years (SD 4.5)  ● Advertisements in newspapers, supermarkets, libraries, pharmacies, general practitioners’ practices, a contact list of individuals who were generally interested in participating in research projects, and lectures for older adults at the University of Zurich  ● Study recruitment dates: 12/2009 - 04/2011  ● Country: Switzerland | 40 // 21 | ● Computer intervention: computer part (about 1/3 of the session time); the patient was provided with a list of questions focusing on negative and positive experiences of his or her past life from childhood until old age  ● 1 male (PhD) and 1 female (Ma) psychologist  with training in psychotherapy and CBT  ● 6 sessions (60-90 minutes), weekly, 6 weeks in total  ● NR  ● NR |
|  |  |  |  |  |  |
| ftf  Beeber (2010) | Beeber LS, et al. Short-term in-home intervention reduces depressive symptoms in Early Head Start Latina mothers of infants and toddlers. Research in nursing & health. 2010;33(1):60-76. | ● Depressive symptoms  ● CES-D | ● Mean: 26 years (SD 5.9)  ● The sample was newly immigrated, Latina mothers with depressive symptoms who had infants or toddlers enrolled in three EHS programs in a new destination area of the southeastern US  ● Study recruitment dates: NR  ● Country: USA | 80 // 39 | ● Peplau’s theory provided the structure for the therapeutic relationship between nurses and mothers guiding delivery of the IPT-based protocol for the intervention  ● English-speaking master’s-prepared psychiatric nurses and project-trained Spanish language interpreters  ● 16 sessions (duration NR), periodicity NR, maximum of 22 weeks in total  ● 11 in-home sessions interspersed with 5 short booster visits by the interpreter alone  ● NR |
| iCBT  Sheeber (2012) | Sheeber LB, et al. Development and pilot evaluation of an Internet-facilitated cognitive-behavioral intervention for maternal depression. Journal of consulting and clinical psychology. 2012;80(5):739-49. | ● Sub-threshold and full syndrome depression  ● CES-D | ● Mean: 31.1 years (SD 5.7)  ● Participants were 70 mothers of children enrolled in Head Start classrooms; Head Start staff gave mothers an invitation letter and the CES-D (the letter provided the informed consent information for the screening)  ● Study recruitment dates: NR  ● Country: USA | 70 // 35 | ● Mom-Net program is an Internet-facilitated, CBT for sub-threshold and full syndrome depression, tailored to economically disadvantaged or rural women with young children; the content foundation for the program was the Coping with Depression Course^2^  ● Coaches, details NR  ● 8 chapters, details NR, most probably 8 weeks in total  ● At home  ● None |
|  |  |  |  |  |  |
| ftf  Prendergast (2001) | Prendergast J, Austin M-PJAP. Early childhood nurse-delivered cognitive behavioural counselling for post-natal depression. Australasian Psychiatry. 2001;9(3):255-9. | ● DSM-IV major or minor depression  ● EPDS | ● Mean: 32.2 years (SD NR)  ● Via regular screening by ECNs of women in the post-natal period using the EPDS  ● Study recruitment dates: NR  ● Country: Australia | 37 // 17 | ● Home-based CBT sessions  ● ECNs; they were only made aware that the women were depressed and needed to be seen more frequently  ● 6 sessions (20-60 minutes), weekly, 6 weeks in total  ● At home or a clinic for mothercraft advice and non-specific emotional support  ● NR |
| iCBT  Milgrom (2016) | Milgrom J, et al. Internet cognitive behavioral therapy for women with postnatal depression: a randomized controlled trial of MumMoodBooster. Journal of medical Internet research. 2016;18(3):e54. | ● Postnatal depression (PND)  ● EPDS | ● Mean: 31.7 years (SD 4.6)  ● Internet campaigns using Google AdWords, Facebook, and Twitter and advertisements in Maternal and Child Health Centers in Melbourne  ● Study recruitment dates: 03/2013 - 07/2014  ● Country: Australia | 43 // 21 | ● The “MumMoodBooster” intervention; designed to encourage optimal engagement and behavior change  ● 3 graduate psychology trainees, 3 clinical psychologists, 1 health psychologist; supported and supervised by 2 senior psychologists  ● 6 sessions (duration NR), periodicity NR, to be completed in 6-12 weeks in total  ● At home  ● None |
|  |  |  |  |  |  |
| ftf  Lustman (1998) | Lustman PJ, et al. Cognitive behavior therapy for depression in type 2 diabetes mellitus: a randomized, controlled trial. Annals of internal medicine. 1998;129(8):613-21. | ● Moderate and high depression in people with diabetes  ●BDI; CES-D | ● Mean and SD NR; Range: 21 - 70 years  ● PCPs working within the Washington University School of Medicine and BJC Healthcare System, St. Louis, Missouri, and it was publicized in various newspapers  ● Study recruitment dates: NR  ● Country: USA | 51 // 25 | ● Patients in the CBT group received individual CBT (behavioral strategies, problem-solving procedures and cognitive techniques)  ● A licensed psychologist  ● 10 sessions (60 minutes), weekly, 10 weeks in total weeks of  ● NR  ● None |
| iCBT  Nobis (2015) | Nobis S, et al. Efficacy of a web-based intervention with mobile phone support in treating depressive symptoms in adults with type 1 and type 2 diabetes: a randomized controlled trial. Diabetes Care. 2015;38(5):776-83. | ● Major depression (DSM) in people with type 2 diabetes  ● CES-D | ● Mean: 51 years (SD NR); Range: 18–79 years  ● Individuals diagnosed with diabetes (collected from a large-scale German health insurance company) were informed of the study and additionally, advertisements were placed in German journals for individuals with diabetes and on social networks such as Facebook  ● Study recruitment dates: NR  ● Country: Germany | 260 // 130 | ● The GET.ON M.E.D. intervention was based on two core evidence-based elements: systematic behavioral activation and problem solving  ● Graduate students or psychologists  ● 6 sessions (up to 45 minutes, option for 2 additional sessions), weekly, 6 weeks in total  ● At home  ● None |
|  |  |  |  |  |  |
| ftf  Murphy (1995) | Murphy GE, et al. Cognitive behavior therapy, relaxation training, and tricyclic antidepressant medication in the treatment of depression. Psychological Reports. 1995;77(2):403-20. | ● Major depressive disorder  ● BDI | ● Mean: 39.8 years (SD 12)  ● Subjects were recruited through local and Washington University news releases  ● Study recruitment dates: NR  ● Country: USA | 37 // 11 | ● CBT according to Beck's et al. model  ● 6 psychologists, 1 social worker, 1 psychiatrist  ● Sessions (total number NR, limited to 50 minutes), once or twice a week for the first 4 weeks and once a week for the remaining 12 weeks, a maximum of 20 sessions in total  ● Washington Medical School (assumed by the reviewer)  ● None |
| iCBT  Selmi (1990) | Selmi PM, et al. Computer-administered cognitive-behavioral therapy for depression. The American Journal of Psychiatry. 1990. | ● Diagnoses for major, minor, or intermittent depressive disorder  ● BDI | ● Mean: 28.9 years (SD 4.89)  ● By newspaper-announcements that included a questionnaire containing 13 depression items from the SCL-90-R and additional items to exclude psychosis  ● Study recruitment dates: NR  ● Country: USA | 36 // 12 | ● The CBT-program was designed by one of the authors; patients were given a short reading assignment and asked to keep a weekly record of their activities and feelings and the program provided a rationale for the assignment and gave explicit instructions for its completion  ● 1 psychological therapist, 1 graduate student in clinical psychology  ● 6 sessions (duration NR), weekly, 6 weeks in total  ● NR  ● None |
|  |  |  |  |  |  |
| ftf  Scott (1997) | Scott C, et al. Acute and one-year outcome of a randomised controlled trial of brief cognitive therapy for major depressive disorder in primary care. The British Journal of Psychiatry. 1997;171(2):131-4. | ● Major depressive disorder (DSM-III)  ● BDI | ● Mean and SD NR; Range: 18-65 years  ● Subjects came from 11 general practices in the northeast of England  ● Study recruitment dates: NR  ● Country: UK | 48 // 24 | ● CT-sessions supplemented by written material; each session included setting agendas, homework tasks, client feedback and selected reading to reinforce the skills being taught  ● 1 therapist with a postgraduate qualification in cognitive therapy, who received regular CT supervision  ● 6 sessions (about 30 minutes), weekly, 6 weeks in total  ● NR  ● None |
| iCBT  Meyer (2009) | Meyer B. Effectiveness of a Novel Integrative Online Treatment for Depression (Deprexis): Randomized Controlled Trial. JOURNAL OF MEDICAL INTERNET RESEARCH. 2009. | ● Depressive symptoms  ● BDI | ● Mean: 34.58 years (SD 11.53)  ● Via advertisements posted on the internet (e.g., by posting brief notices on depression-related internet forums in Germany, given the permission of the forum administrators)  ● Study recruitment dates: 02/2007 - 06/2008  ● Country: Germany | 396 // 320 | ● The Web-based intervention with ten content modules representing different psychotherapeutic approaches, plus one introductory and one summary module  ● NR  ● 10 modules (10-60 minutes), weekly, 9 weeks in total  ● NR  ● None |
|  |  |  |  |  |  |
| ftf  Castonguay (2004) | Castonguay LG, et al. Integrative cognitive therapy for depression: a preliminary investigation. Journal of Psychotherapy Integration. 2004;14(1):4-20. | ● DSM-IV criteria for MDD  ● BDI | ● Mean (total sample): 38.8 years, (SD 10.9)  ● By advertisements  ● Study recruitment dates: NR  ● Country: USA | 28 // 14 | ● CT following the guidelines of Beck et al.’s treatment manual, unless problems in the therapeutic relationship emerged during treatment  ● 7 graduate students in clinical psychology  ● 12-20 sessions of individual therapy (duration NR), biweekly for the first 2 - 4 weeks, and weekly for the rest of the treatment (mean of 17.3 weeks in total)  ● At the Pennsylvania State University  ● NR |
| iCBT  Ruwaard (2009) | Ruwaard J, et al. Standardized web-based cognitive behavioural therapy of mild to moderate depression: a randomized controlled trial with a long-term follow-up. Cognitive behaviour therapy. 2009;38(4):206-21. | ● Mild to moderate depression  ● BDI | ● Mean: 42 years (SD 10)  ● An article in a national Dutch newspaper announcing the study  ● Study recruitment dates: NR  ● Country: Netherlands | 54 // 36 | ● CT and behavioral activation  ● 12 graduate-level clinical psychologists and 6 therapists of the Jellinek Mentrum Mental Health Care Organization Amsterdam  ● 8 phases (120-240 minutes per week to complete the assignments), periodicity NR, 11 weeks in total (assumed by the reviewer)  ● NR  ● None |
|  |  |  |  |  |  |
| ftf  Mynors-Wallis (1995) | Mynors-Wallis, et al. Randomised controlled trial comparing problem solving treatment with amitriptyline and placebo for major depression in primary care. Bmj. 1995;310(6977):441-5. | ● Major depression  ● BDI (as well as HAMD) | ● Mean: 37.3 years (SD 12.8)  ● From 26 general practitioners working in 15 local practices  ● Study recruitment dates: NR  ● Country: UK | 91 // 30 | ● The stages of problem solving were explained to the patient by reference to the chosen problem and in subsequent sessions further problems were dealt with in the same way  ● 1 psychiatrist (experienced in problem solving), 2 general practitioners (trained in problem solving and in a standardized method of drug administration)  ● 6 sessions (first one 60 minutes, others 30 minutes), periodicity NR, maximum of 12 weeks in total  ● In the patient's home or local health center  ● NR |
| iCBT  Vernmark (2010) | Vernmark K, et al. Internet administered guided self-help versus individualized e-mail therapy: A randomized trial of two versions of CBT for major depression. Behaviour research and therapy. 2010;48(5):368-76. | ● Major depression (DSM-IV)  ● BDI | ● Mean: 37.2 years (SD 13.0)  ● By means of newspaper articles in national and regional papers, a national radio interview regarding the study, posters at the University campus, and information on various web pages  ● Study recruitment dates: NR  ● Country: Sweden | 88 // 29 | ● The self-help program consisted of 7 text modules totaling 114 pages, including exercises; it included an introduction to CBT, depression from a CBT-perspective with a behavioural focus, behavioural activation, cognitive restructuring, sleep management, defining goals/values and relapse prevention.  ● 6 psychology M.Sc. students who had completed their clinical training  ● 7 modules, duration and periodicity NR, 7 weeks in total  ● NR  ● None |
|  |  |  |  |  |  |
| ftf  Freedland (2009) | Freedland KE, et al. Treatment of depression after coronary artery bypass surgery: a randomized controlled trial. Archives of general psychiatry. 2009;66(4):387-96. | ● DSM-IV criteria for a current major or minor depressive episode  ● BDI | ● Mean: 62 years (SD 11)  ● Patients aged 21 years or older who had undergone CABG surgery within the past year at a hospital.  ● Study recruitment dates: NR  ● Country: USA | 123 // 41 | ● CBT (treatment manuals were standard CBT texts)  ● 2 clinical psychologists and a clinical social worker  ● minimum of 12 sessions (50-60 minutes), weekly, 12 weeks in total  ● NR  ● NR |
| iCBT  van Bastelaar (2011) | van Bastelaar KM, et al. Web-based depression treatment for type 1 and type 2 diabetic patients: a randomized, controlled trial. Diabetes Care. 2011;34(2):320-5. | ● Depression in people with diabetes  ● CES-D | ● Mean: 48 years (SD 12)  ● By advertisements in various general and diabetes specific media  ● Study recruitment dates: 07/2008 - 09/2009  ● Country: the Netherlands | 255 // 125 | ● Written and spoken information and videos including diabetes-specific topics in the eight scheduled lessons of the online ‘Coping with depression’ course^2^.  ● Certified health psychologists, details NR  ● 8 lessons, duration and periodicity NR, 8 weeks in total  ● NR, at home (assumed by the reviewer)  ● NR |
|  |  |  |  |  |  |
| ftf  Rodriguez (2004) | Rodríguez JL, et al. Estudio doble ciego con antidepresivo, psicoterapia breve y placebo en pacientes con depresion leve a moderada. Salud mental. 2004;27(5):53-61. | ● Mild to moderate depression (DSM-IV and ICD-10)  ● HAMD | ● Mean: 31.9 years (SD 7.9)  ● From outpatients attending the Centro de Salud Castro Villagrana  ● Study recruitment dates: NR  ● Country: Mexico | 40 // 10 | ● Bellak's psychotherapy without pharmacotherapy  ● Psychotherapeutic nurse or clinical psychologist, details NR  ● sessions (number NR) (50 minutes), weekly, 90 consecutive days in total  ● At the Centro de Salud Castro Villagrana  ● None |
| iCBT  Klein (2016) | Klein JP, et al. Effects of a Psychological Internet Intervention in the Treatment of Mild to Moderate Depressive Symptoms: Results of the EVIDENT Study, a Randomized Controlled Trial. Psychotherapy and psychosomatics. 2016;85(4):218-28. | ● Mild to moderate depressive symptoms  ● PHQ-9 | ● Mean: 45 years (SD NR); Range: 18-65 years  ● Inpatient and outpatient medical and psychological clinics, online forums for depression, health insurance companies and the media (e.g. newspaper and radio)  ● Study recruitment dates: 08/2012 - 12/2013  ● Country: Germany | 1013 // 509 | ● The Internet intervention (a CBT-based programme called “Deprexis”^1^), which consists modules covering content that is broadly consistent with CBT (e.g., cognitive restructuring, behavioural activation, acceptance and mindfulness, problem solving), plus one summary module  ● NR  ● 10 modules (duration NR), weekly, 12 weeks in total  ● NR, at home (assumed by the reviewer)  ● CAU |
|  |  |  |  |  |  |
| ftf  Allart-van Dam (2003) | Allart-van Dam E, et al. The coping with depression course: short-term outcomes and mediating effects of a randomized controlled trial in the treatment of subclinical depression. Behavior Therapy. 2003;34(3):381-96. | ● Subclinical depressive symptoms  ● BDI | ● Mean: 45.5 years (SD 9.9); Range: 18-65 years  ● By means of a call in local newspapers and on local television.  ● Study recruitment dates: NR  ● Country: Netherlands | 110 // 68 | ● The Dutch version of the “Coping With Depression^”^ course ^2^; a cognitive behavioral intervention that teaches the participants techniques to influence their mood and to enhance their coping skills for problems related to their depressive symptoms  ● 2 psychologists and 2 graduate students of psychology  ● 12 sessions (120 minutes); weekly, 12 weeks in total  ● NR  ● CAU by GP |
| iCBT  Moritz (2012) | Moritz S, et al. A randomized controlled trial of internet-based therapy in depression. Behaviour research and therapy. 2012;50(7-8):513-21. | ● Elevated depression symptoms  ● BDI | ● Mean: 38.00 years (SD 10.76)  ● On several internet support networks devoted to depression (they disseminate information about the disorder and offer a platform for people with depression to exchange opinions and advice)  ● Study recruitment dates: NR  ● Country: Germany | 208 // 105 | ● Deprexis^1^; the program encompasses ten content modules with a strong focus on evidence-based cognitive-behavioral techniques, either from its so-called first (behavior-oriented), second (cognitive-oriented), or third wave (emotion-oriented, e.g., mindfulness and acceptance)  ● NR  ● 10 modules (10-60 minutes), weekly, at least 10 weeks in total  ● NR, at home (assumed by the reviewer)  ● CAU |
|  |  |  |  |  |  |
| ftf  Pace (1993) | Pace TM, Dixon DNJJoCP. Changes in depressive self-schemata and depressive symptoms following cognitive therapy. Journal of Counseling Psychology. 1993;40(3):288. | ● Mildly and moderately depressed college students' depressive symptoms and depressive self-schemata  ● BDI | ● Mean: 22.54 years (SD 4.68)  ● From a large state university in the Midwest  ● Study recruitment dates: NR  ● Country: USA | 74 // 31 | ● Individual sessions of CT; the treatment followed a protocol adapted from Beck et al.  ● 7 graduate students  ● 6-8 sessions (45 minutes), once or twice a week, 4 - 7 weeks in total  ● University  ● None |
| iCBT  Clarke (2009) | Clarke G, et al. Randomized effectiveness trial of an Internet, pure self-help, cognitive behavioral intervention for depressive symptoms in young adults. Cognitive behaviour therapy. 2009;38(4):222-34. | ● Depression symptoms  ● PHQ-8 | ● Mean: 22.6 years (SD 2.3)  ● The study was conducted in a health maintenance  organization (HMO) with more than 450,000 members in the Northwest United States  ● Study recruitment dates: 2005  ● Country: USA | 160 // 83 | ● A pure self-help site (unattended in that it is not staffed by live personnel); it provides self-guided, interactive behavioral and cognitive therapy tutorials to help users overcome depression and offers significant interactivity and tailoring that distinguishes it from static, traditional bibliotherapy sites that deliver identical content to every user  ● NR  ● Details NR; from enrollment to the final assessment 32 weeks in total  ● NR, at home (assumed by the reviewer)  ● CAU |
|  |  |  |  |  |  |
| ftf  Strauman (2006) | Strauman TJ, et al. Self-system therapy as an intervention for self-regulatory dysfunction in depression: a randomized comparison with cognitive therapy. Journal of Consulting and Clinical psychology. 2006;74(2):367. | ● DSM–IV; criteria for major depressive disorder or dysthymic disorder  ● BDI | ● Mean: 39.4 years (SD 14.2); Range: 19-72 years  ● Via announcements on local television news broadcasts and in newspapers, intake at a university psychiatric clinic or women’s health clinic, or referral from a university counseling center  ● Study recruitment dates: 1999 - 2000  ● Country: USA | 45 // 21 | ● SST is a new therapy based on regulatory focus theory; treatment was ended when the therapist indicated the final task of the treatment (discussion of relapse prevention) was completed  ● 9 therapists (2 faculty clinical psychologists, 4 clinical psychology postdoctoral fellows, and 3 clinical psychology predoctoral interns)  ● At least 12 sessions (duration NR); weekly, then at least once every other week, weeks in total NR  ● University psychiatric clinic  ● NR |
| iCBT  Rosso (2017) | Rosso IM, et al. Internet-based cognitive behavior therapy for major depressive disorder: A randomized controlled trial. Depression and anxiety. 2017;34(3):236-45. | ● Major depressive disorder  ● PHQ-9 | ● Mean and SD NR; Range: 18–45 years  ● Internet advertisements and community fliers  ● Study recruitment dates: 10/2012 -08/2015  ● Country: USA | 77 // 37 | ● Modified version of the Sadness Program^3^, a technician-assisted iCBT program developed at the UNSW; modifications involved language adaptations and minor content alterations for relevance to American culture (e.g., providing MDD prevalence rates for the U.S. rather than Australia)  ● Trained bachelor-level research assistant, details NR  ● 6 lessions (duration NR), weekly, 10 weeks in total  ● NR, at home (assumed by the reviewer)  ● NR |
|  |  |  |  |  |  |
| ftf  Goldman (2006) | Goldman RN, et al. The effects of adding emotion-focused interventions to the client-centered relationship conditions in the treatment of depression. Psychotherapy Research. 2006;16(5):537-49. | ● DSM IV criteria for major depressive disorder (32% clients were diagnosed with an Axis II personality disorder)  ● BDI | ● Mean: 39.5 years (SD 9.71)  ● Local media, including television programs, radio announcements, and local newspapers  ● Study recruitment dates: NR  ● Country: Canada | 38 // 19 | ● CCT; treatment followed the manual for relational CCT and therapists in this condition adopt the three fundamental relational attitudes of empathy, positive regard, and congruence  ● 3 of the therapists were licensed clinical psychologists, 2 were doctorate-level clinical psychologists, 9 were advanced doctoral students in clinical psychology  ● 16 - 20 sessions (duration NR), weekly, total duration NR  ● At a Psychotherapy Research Clinic  ● None |
| iCBT  Williams (2015) | Williams AD, et al. Positive imagery cognitive bias modification (CBM) and internet-based cognitive behavioral therapy (iCBT): a randomized controlled trial. Journal of Affective Disorders. 2015;178:131-41. | ● Major depression  ● BDI-II | ● Mean: 43.94 years (SD 10.80)  ● Via the research arm of a not-for-profit clinical and research unit affiliated with St.Vincent's Hospital and the University of New South Wales  ● Study recruitment dates: NR  ● Country: Australia | 121 // 60 | ● The Sadness Program^3^ (evaluated in four previous trials, an effectiveness study, and delivered as part of a routine clinical service in Australia since 2010); online lessons representing best practice CBT, as well as regular homework assignments and access to supplementary resources  ● NR  ● 6 lessions (duration NR), weekly, 10 weeks in total  ● NR, at home (assumed by the reviewer)  ● NR |
|  |  |  |  |  |  |
| ftf  Watson (2003) | Watson JC, et al. Comparing the effectiveness of process-experiential with cognitive-behavioral psychotherapy in the treatment of depression. Journal of consulting and clinical psychology. 2003;71(4):773-81. | ● Major depression (DSM-IV)  ● BDI | ● Mean: NR; Range: 21–65 years (SD = 10.82)  ● Recruited through advertisements  ● Study recruitment dates: NR  ● Country: Canada | 93 // 45 | ● The CBT protocol was conducted according to the cognitive therapy treatment for depression outlined by Beck et al. The treatment was primarily a cognitive therapy with some behavioral components, such as the recording of daily activities and behavioral experiments.  ● 13 master’s or doctoral candidates in counseling psychology, 2 psychologists  ● 16 sessions (60 minutes), weekly, 16 weeks in total  ● An outpatient clinic affiliated with a large metropolitan university in Southern Ontario  ● NR |
| iCBT  García (2010) | García AV, et al. Internet treatment for depression: a randomized controlled trial comparing clinician vs. technician assistance. PLoS one. 2010;5(6). | ● Major depressive disorder  ● PHQ | ● Mean: 40years, (SD 12.33)  ● Via a website (www.virtualclinic.org.au) providing information about common mental disorders including depression, and a link to apply online to join a research treatment program.  ● Study recruitment dates: 06 - 07/2009  ● Country: Australia | 127 // 46 | ● The Sadness program^3^ (iCBT program) consists of 6 online lessons, printable summary and homework assignments, automatic emails, and additional resource documents. The Clinician-Assisted Treatment group participants had weekly email or telephone contact with the clinician and access to an online discussion forum where they could post questions to the clinician about the program content.  ● Qualified and registered psychiatrist  ● 6 lessons (duration NR), 1 lesson every  7–10 days, 8 weeks in total  ● NR, at home (assumed by the reviewer)  ● NR |
|  |  |  |  |  |  |
| ftf  Sethi (2013) | Sethi S. Treating youth depression and anxiety: a randomised controlled trial examining the efficacy of computerised versus face-to-face cognitive behaviour therapy. Australian Psychologist. 2013;48(4):249-57. | ● Depression mild to moderate (scores from 10 - 20) and/or generalized anxiety  ● DASS 21 | ● Mean: 20.3 (SD 1.39)  ● Recruited from community youth centers, located in Sydney’s North and Inner West regions. The management of 28 local youth centers were contacted, and 13 centers showed interest in the study.  ● Study recruitment dates: NR  ● Country: Australia | 89 // 21 | ● Each session focused on the treatment of depression and/or anxiety using the cognitive behavioural model of therapy. The face-to-face CBT approach in this study is based on Beck’s conceptualization of CBT, and the therapist used a CB manual developed by Menzies as part of an advanced CBT workshop.  ● Registered psychologist  ● 5 sessions (60 minutes), weekly, 5 weeks in total  ● local community centers or at the university campus  ● NR |
| iCBT  Sethi (2013) | Sethi S. Treating youth depression and anxiety: a randomised controlled trial examining the efficacy of computerised versus face-to-face cognitive behaviour therapy. Australian Psychologist. 2013;48(4):249-57. | ● Depression mild to moderate (scores from 10 - 20) and/or generalized anxiety  ● DASS 21 | ● Mean: 20.78, (SD 1.22)  ● Recruited from community youth centers, located in Sydney’s North and Inner West regions. The management of 28 local youth centers were contacted, and 13 centers showed interest in the study.  ● Study recruitment dates: NR  ● Country: Australia | 89 // 23 | ● MoodGYM (automated online CBT intervention, created by the Australian National University); based on cognitive behavioural principles including cognitive restructuring, pleasant events scheduling, and interpersonal problem solving.  ● Registered psychologist  ● 5 sessions (60 minutes), weekly, 5 weeks in total  ● local community centers or at the university campus  ● NR |
|  |  |  |  |  |  |
| ftf  Preschl (2011)^4^ | Preschl B, et al. The working alliance in a randomized controlled trial comparing online with face-to-face cognitive-behavioral therapy for depression. BMC psychiatry. 2011;11(1):189. | ● Mild-moderate  ● BDI | ● Mean: 38.3 years (SD 11.9)  ● Through advertisements in newspapers, the depression website of the university, local internet news forums, and depression self-help groups, advertisements in supermarkets and pharmacies, and local press releases.  ● Study recruitment dates: 11/2008 - 02/2010  ● Country: Switzerland | 53 // 28 | ● The face-to-face condition follows an evidence-based short-term CBT treatment manual for depression by Beck and colleagues.  ● 6 female psychologists and psychotherapists  ● 8 sessions (60 minutes), weekly, 8 weeks in total  ● Department of Psychopathology and Clinical Intervention at the University of Zurich  ● NR |
| iCBT  Preschl (2011)^4^ | Preschl B, et al. The working alliance in a randomized controlled trial comparing online with face-to-face cognitive-behavioral therapy for depression. BMC psychiatry. 2011;11(1):189. | ● Mild-moderate  ● BDI | ● Mean: 34.9 years (SD 9.5)  ● Through advertisements in newspapers, the depression website of the university, local internet news forums, and depression self-help groups, advertisements in supermarkets and pharmacies, and local press releases.  ● Study recruitment dates: 11/2008 - 02/2010  ● Country: Switzerland | 53 // 25 | ● The CBT treatment manual for depression was adapted for use as an internet-based intervention; the treatment consisted of structured writing and homework assignments (e.g., behavioral analysis of depressive symptoms, activity diaries, cognitive restructuring worksheets) based on the CBT approach and on the written disclosure procedure developed by Pennebaker and colleagues and follows an evidence-based short-term CBT treatment manual for depression by Beck and colleagues  ● 6 female psychologists and psychotherapists  ● 8 sessions (45 minutes), weekly, 8 weeks in total  ● NR, at home (assumed by the reviewer)  ● NR |

***Abbreviations***

**AGECAT** = Automated Geriatric Examination for Computer Assisted Taxonomy**; AOD** = alcohol and other drugs; **BA** = Behavioral Activation; **BDI (I/II)** = Beck Depression Inventory (I/II); **CABG surgery** = coronary bypass artery; **CAU** = care as usual; **CBDI** = Chinese versions of the Beck Depression Inventory; **CBT** = cognitive behavioral therapy; **CCBT** = computerized CBT; **CCT** = client-centered therapy; **CES-D** = Center for Epidemiological Studies**-**Depression scale; **CGI** = Clinical Global Impression of severity and improvement; **CNT** = Concrete training guided self-help; **CT** = cognitive therapy; **DSM-IV** = diagnostic and statistical manual of mental disorders, version IV; **ECN** = Early Childhood Nurse; **EHS** = Early Head Start; **EPDS** = Edinburgh Postnatal Depression Scale; **ftf** = face-to-face; **GP** = general practitioner; **HADS/HAD-S** = Hospital Anxiety and Depression Scale; **HAD-D** = depression separately in the Hospital Anxiety and Depression Scale; **HAMD (/HDS/HRDS/HRSD)** = Hamilton rating scale for depression; **ICD** = International Statistical Classification of Diseases and Related Health Problems; **iCBT** = internet-based cognitive behavioral therapy; **IPT** = interpersonal therapy; **MADRS-S** = Montgomery-Åsberg Depression Rating Scale; **MDD** = Major depressive disorder; **MI** = motivational interviewing; **NR** = not reported; **PCPs** = primary care physicians; **PCT** = person-centered therapy; **PST** = problem solving therapy; **SCID/SCID-RV** = structured clinical interview for DSM-IV/V; **SCL-90-R** = Symptom Checklist-90-R; a report inventory that is used to measure the distress and other psychological symptoms; **SD** = standard deviation; **SPSP** = Short-Term Psychodynamic Supportive Psychotherapy; **SET** = supportive expressive therapy; **SST** = self-system therapy; **TAU** = treatment as usual; **RCT** = randomized controlled trial; **UNSW** = University of New South Wales, Sydney (Australia) **UK** = United Kingdom

***Footnotes***

**^1^ Deprexis:** The Web-based intervention consists of 10 modules representing different psychotherapeutic approaches, plus one introductory and one summary module, each of which can be completed in 10 to 60 minutes, depending on the user’s reading speed, interest, motivation, and individual path through the program. Modules are organized as simulated dialogues in which the program explains and illustrates concepts and techniques, engages the user in exercises, and continuously asks users to respond by selecting from response options. Subsequent content is then tailored to the users’ responses, resulting in a simulated conversational flow. All modules are accompanied by illustrations (e.g., drawings, photographs, flash animations).

(https://www.jmir.org/2009/2/e15/ // https://de.deprexis.com/)

**^2^ Coping with depression**: The “Coping with Depression” course (CWD). The first version of this treatment was developed in the late 1970s by Prof. Lewinsohn and his associates. Since the publication of the manual (Lewinsohn, Antonuccio, Breckenridge, & Teri, 1984), many other researchers and practitioners have used this course in the treatment, prevention and relapse prevention of depression in many target groups. The course materials have been translated into several languages, and the course is actively used in many countries, including the United States, Canada, Germany, Finland, Mexico, The Netherlands and Perú. The CWD is now one of the most widely available psychological treatments for depression worldwide and is one of the best studied psychological treatments of depression in general.

(https://www.sciencedirect.com/science/article/pii/S027273580900049X?casa_token=VpF7KJzgeRsAAAAA:mm4MB5nVMjsAgxSEmL1cn5L-fE6Z9ZpdYVA6ckiTedJwXSZFb6Pa6mEln1MbkDA_RMwuFlU#bbib5)

**^3^ Sadness Program**: https://www.c4tbh.org/program-review/the-sadness-program/

**^4^** This study was described in two reports: Preschl (2011) and Wagner (2011)
