# Supplementary material for: Comparability of Patients in Trials of eHealth and Face-to-Face Psychotherapeutic Interventions for Depression: Meta-synthesis
Source: J Med Internet Res. 2022 Sep 14;24(9):e36978. doi: 10.2196/36978 (PMC9520399; doi:10.2196/36978)
Supplement: Multimedia Appendix 3 [file jmir_v24i9e36978_app3.docx]

**Appendix 3. Descriptive characteristics of the included studies**

|  |  |  |
| --- | --- | --- |
| **Characteristics** | **Internet-studies** | **Face-to-face-studies** |
| **Included studies (n)** | 29 | 29 |
| **Depressive symptoms measurement** |  |  |
| BDI | 20 | 20 |
| HAMD // HAD-D/S | 1 | 3 |
| CES-D | 5 | 3 |
| EPDS | 2 | 2 |
| Other (DASS-21) | 1 | 1 |
| **Diagnosis** |  |  |
| Mild to moderate depression | 13 | 9 |
| Major depression | 11 | 13 |
| Post-natal depression | 2 | 2 |
| Type of depression not specified | 3 | 5 |
| **Year** |  |  |
| 1990-1999 | 1 | 5 |
| 2000-2009 | 6 | 11 |
| 2010-2017 | 22 | 13 |
| **Country** |  |  |
| USA | 4 | 9 |
| Australia | 6 | 4 |
| Netherlands | 5 | 3 |
| UK | 1 | 7 |
| Germany | 5 | 0 |
| Sweden | 4 | 0 |
| Switzerland | 3 | 1 |
| Other | 1 | 5 |
| **N of participants per intervention group** |  |  |
| 1-60 participants | 17 | 24 |
| 61-120 participants | 6 | 5 |
| >120 participants | 6 | 0 |

**BDI:** Beck Depression Inventory, **HAMD:** Hamilton Rating Scale for Depression, **HAD-D/S:** Hospital Anxiety and Depression Scale, **CES-D:** Centre for Epidemiologic Studies-Depression Scale, **EPDS:** Edinburgh Postnatal Depression Score, **DASS-21:** Depression Anxiety Scale, **DM2:** Diabetes mellitus type II
