# Supplementary material for: Comparability of Patients in Trials of eHealth and Face-to-Face Psychotherapeutic Interventions for Depression: Meta-synthesis
Source: J Med Internet Res. 2022 Sep 14;24(9):e36978. doi: 10.2196/36978 (PMC9520399; doi:10.2196/36978)
Supplement: Multimedia Appendix 4 [file jmir_v24i9e36978_app4.docx]

**Appendix 4. Meta-analyses of baseline characteristics**

| Outcome | Meta-analysis: all studies  (RE model) | | | Meta-analysis: iCBT  (RE model) | | | Meta-analysis: face-to-face CBT  (RE model) | | | | Subgroup analyses^a^ | |
| --- | --- | --- | --- | --- | --- | --- | --- | --- | --- | --- | --- | --- |
|  | Estimate  (95% CI) | Nr. Studies  Nr. Participants | I^2^ | Estimate  (95% CI) | Nr. Studies  Nr. Participants | I^2^ | Estimate  (95% CI) | Nr. Studies  Nr. Participants | | I^2^ | Difference  (95% CI)^b^ | P-value |
| **Age** | 40.62  (36.75 to 44.49) | 57  3630 | 99.6% | 39.98  (35.22 to 44.74) | 29  2574 | 99.7% | 41.81  (35.21 to 48.54) | | 28  1056 | 99.5% | - 1.89  (- 10.08 to 6.29) | .65 |
| **Gender (% women)** | 71.5%  (68.3% to 74.4%) | 58  3846 | 70.2% | 72.9%  (69.2 to 76.4%) | 29  2575 | 66% | 68.2%  (62.2 to 73.7%) | | 29  1271 | 71.1% | **-** | .16 |
| **Education (% higher education)** | 82.7%  (77.3% to 87.0%) | 37  2590 | 85.9% | 84.1%  (77.8 to 88.8%) | 22  1801 | 86.4% | 79.2%  (67.4 to 87.5%) | | 15  789 | 85.2% | **-** | .38 |
| **Living area (% metropolitan area)** | 99%  (96.1% to 99.7%) | 4  397 | < .001% | 99.5%  (96.2 to 99.9%) | 2  345 | 36.9% | 98.1%  (88.0 to 99.7%) | | 2  52 | < .001% | **-** | .38 |
| **Depression score (standardized to 0-100-point scale) ^c^** | 42.50  (38.60 to 42.39) | 57  3601 | 98.2% | 41.34  (37.37 to 45.31) | 29  2581 | 98.6% | 42.25  (38.09 to 42.41) | | 28  1020 | 96.5% | 1.10  (-3.43 to 5.61) | .64 |
| **Depression duration (years)** | 3.7  (2.2 to 5.2) | 6  191 | 93.1% | 10.0  (5.6 to 14.4) | 1  36 | .001% | 2.8  (1.2 to 4.4) | | 5  155 | 89.2% | 7.19  (2.53 to 11.84) | **.002** |
| **History of depression (%)** | 62.4%  (52.4% to 71.4%) | 20  1116 | 89.1% | 56.6%  (39.0 to 72.7%) | 10  774 | 93.3% | 65.1%  (53.1 to 75.5%) | | 10  342 | 73.3% | **-** | .42 |
| **Previous depression treatment (%) ^d^** | 29.6%  (23.1% to 37.1%) | 15  1203 | 80.4% | 24.8%  (18.0 to 33.1%) | 8  908 | 75.2% | 42.0%  (28.3 to 57.1%) | | 7  303 | 80.9% | **-** | **.035** |
| **Actual antidepressant medication (%)** | 30.1%  (21.7% to 40.0%) | 26  1842 | 88.7% | 33.1%  (23.6 to 44.2%) | 13  1419 | 91.3% | 14.8%  (5.0 to 36.6%) | | 13  423 | 85.3% | **-** | .11 |
| **Actual physical comorbidity (%)** | 99.2%  (97.0% to 99.8%) | 4  320 | .001% | 99.6%  (97.3 to 99.9%) | 2  254 | .001% | 98.5%  (90.0 to 99.8%) | | 2  66 | .001% | **-** | .33 |
| **Actual mental comorbidity (%)** | 70.8%  (44.9% to 87.8%) | 10  328 | 86.1% | 73.8%  (39.2 to 92.5%) | 5  132 | 84.6% | 66.9%  (28.7 to 91.1%) | | 5  196 | 89.7% | **-** | .77 |
| **Study drop-outs (%)** | 17.8%  (13.8% to 22.7%) | 48  2865 | 87.3% | 19.5%  (14.1 to 26.4%) | 24  1878 | 89.5% | 15.4%  (10.1 to 22.7%) | | 24  987 | 83.4% | **-** | .36 |
| **Quality of life (standardized to 0-100-point scale)** | 45.48  (35.06 to 55.89) | 11  994 | 99.4% | 48.11  (36.59 to 59.62) | 9  904 | 99.3% | 33.61  (9.15 to 58.07) | | 2  90 | 98.5% | 14.50  (-12.54 to 41.53) | .29 |
| **Proficiency with computers** | - | **-** | **-** | **-** | **-** | **-** | **-** | | **-** | **-** | **-** | **-** |
| **Having children (%)** | 98.6%  (95.8 to 99.6%) | 6  300 | .001% | 99.0%  (95.3 to 99.8) | 3  221 | .001% | 98.1%  (91.0 to 99.6%) | | 3  79 | .001% | **-** | .55 |
| **Family status (% living alone)** | 40.6%  (34.6% to 47.0%) | 40  256 | 86.6% | 38.3%  (30.8 to 46.5%) | 20  1795 | 88.2% | 44.2%  (34.5 to 54.4%) | | 20  768 | 83.3% | **-** | .37 |
| **Employment (% being employed)** | 56.4%  (48.0% to 64.4%) | 26  1932 | 86.9% | 59.4%  (47.9 to 69.9%) | 13  1413 | 90.6% | 53.0%  (40.9 to 64.8%) | | 13  519 | 80.9% | **-** | .45 |

*Abbreviations*: **-**: no data. **BDI**: Beck’s Depression Inventory. **CBT**: cognitive-behavioural therapy. **CES-D**: Center for Epidemiologic Studies Depression Scale. **DASS-21**: Depression, Anxiety and Stress Scale-21. **ED**: Emergency department. **EPDS**: Edinburgh Postnatal Depression Scale. **HDRS**: Hamilton Depression Rating Scale (also known as HAM-D). **iCBT**: eHealth CBT. **Nr**: Number. **RCT**: Randomised clinical controlled trial. **RE**: random-effects model.

P-value ≤ 0.05 is shown in bold type.

^a^ Degrees of freedom (df) = 1

^b^ CI 95% for the difference in prevalences was not as there is no meaningful way to compute it.

^c^ Subgroup analyses for depression measured with individual scores: BDI (P value= .996); DASS-21 (P value=.038); CES-D (6 studies, P value= .865), EPDS (4 studies, P value= .338), and HDRS (6 studies, P value= .069).

^d^ Proportion of patients (%) having received any kind of treatment for depression, that is, psychotherapy, antidepressants, or both.
